# Assessment and mapping of noise pollution in recreation spaces using geostatistic method after COVID-19 lockdown in Turkey

**DOI:** 10.1007/s11356-024-33434-3

**Published:** 2024-04-29

**Authors:** Rifat Olgun, Nihat Karakuş, Serdar Selim, Buket Eyileten

**Affiliations:** 1 Vocational School of Serik G-S. Sural, Akdeniz University, Antalya, 07500 Turkey; 2 The Design School, Arizona State University, Tempe, 85287 USA; 3 Faculty of Science, Akdeniz University, 07058 Antalya, Turkey; 4 Institute of Science, Akdeniz University, 07058 Antalya, Turkey

**Keywords:** Urban planning, Urban open green space, Noise pollution, Noise mapping, COVID-19 lockdown, Geostatistical interpolation

## Abstract

Increased use of recreational areas after the lifting of COVID-19 pandemic restrictions has led to increased noise levels. This study aims to determine the level of noise pollution experienced in recreational areas with the increasing domestic and international tourism activities after the lifting of pandemic lockdowns, to produce spatial distribution maps of noise pollution, and to develop strategic planning suggestions for reducing noise pollution in line with the results obtained. Antalya-Konyaaltı Beach Recreation Area, the most important international tourism destination of Turkey, is determined as the study area. To determine the existing noise pollution, 31 measurement points were marked at 100 m intervals within the study area. Noise measurements were taken during the daytime (07:00–19:00), evening (19:00–23:00), and nighttime (23:00–07:00) on weekdays (Monday, Wednesday, Friday) and weekends (Sunday) over 2 months in the summer when the lockdown was lifted. In addition, the sound level at each measurement point was recorded for 15 min, while the number of vehicles passing through the area during the same period was determined. The database created as a result of measurements and observations was analyzed using statistical and geostatistical methods. After the analysis of the data, it was found that the co-kriging-stable model showed superior performance in noise mapping. Additionally, it was revealed that there is a high correlation between traffic density and noise intensity, with the highest equivalent noise level (Leq) on weekdays and weekend evenings due to traffic and user density. In conclusion, regions exposed to intense noise pollution were identified and strategic planning recommendations were developed to prevent/reduce noise sources in these identified regions.

## Introduction

The escalation of the global population, rapid urbanization, and advancements in industry and transportation contribute to the emergence of various forms of pollution, encompassing air, water, soil, and noise (Alimohammadi et al. 2013; Oyedepo 2013; Munir et al. 2021). These pollutants that tend to be more effective in urban areas demonstrate a gradual increase over time attributable to anthropogenic influences (Margaritis and Kang 2017a; Rocha et al. 2017; Lagonigro et al. 2018; Hien et al. 2020). Recent study findings suggest that noise pollution will become one of the prominent issues among urban challenges. Highlighted in the study conducted by the European Noise Directive (2017), the adverse impact of noise pollution on the quality of life in both advanced and developing nations is underscored. Additionally, it is stated that this phenomenon negatively impacts human health, thereby constituting a significant health concern such as headaches, hypertension, cardiac issues, hearing difficulties, attentional disturbances, restlessness, and cognitive impairments (Arana et al. 2010; Oyedepo and Saadu 2010; Dal and Yugruk Akdag 2011; Oyedepo et al. 2019; Kalawapudi et al. 2020; Sonaviya and Tandel 2020; de Lima Andrade et al. 2021). The World Health Organization (WHO), taking into consideration the health implications of noise, has formally acknowledged ambient acoustic disturbances as an adverse pollutant impacting human health (WHO 2011; Begou and Kassomenos 2021). According to the European Environment Agency, approximately 82 million people are exposed to sound levels above 55 dB, which has a negative impact on human health (Khan et al. 2021).

In today’s world, the development of environmental noise in urban areas is primarily driven by transport vehicles (such as highway noise, railway noise, and airport noise), along with construction and industrial activities (Licitra et al. 2011, 2012; Bozkurt 2021; Salem 2021). Another significant noise source in urban areas is recreational activities (Jakovljević et al. 2006). Upon examining noise pollution resulting from recreational activities, it is observed that urban tourism activities amplify noise levels, thus it is assessed as a factor augmenting the residents’ discomfort levels. Over the past years, particularly in nations with coastal proximity, problems related to this issue have been escalating. Therefore, study endeavors are being undertaken in the field of recreational noise pollution (Victoria State Government 2016; Akbulut Çoban et al. 2018; Ottoz et al. 2018; Petri et al. 2021).

Researchers conduct scientific studies to mitigate or prevent noise pollution, and various institutions and organizations analyze noise maps and formulate comprehensive action plans. Collaborative initiatives between researchers and institutions form a platform, that leads to the development of various strategies in response to study findings (Iglesias-Merchan et al. 2015; Bunn and Zannin 2016; Licitra et al. 2016, 2017; Ruiz-Padillo et al. 2016; Gagliardi et al. 2017; Ozkurt et al. 2018; Tezel et al. 2019). Nevertheless, the acoustic environment in a city or region can vary temporally and spatially depending on factors such as the city’s architectural configuration, the structure of the city’s road network, weather conditions, and existing vegetation (Torija et al. 2011; Maruyama et al. 2013; Prieto Gajardo and Barrigón Morillas 2015). Hence, diverse methodologies and tools are available to detect and map noise pollution (Lee et al. 2014; Vogiatzis and Remy 2014; Gulliver et al. 2015). Noise mapping studies are conducted utilizing Geographic Information System (GIS) based software, notably widely used in the majority of European Union (EU) countries as well as in nations like Turkey, Japan, and the USA (Cai et al. 2015; Harman et al. 2016). In conjunction with the mentioned GIS-based software, interpolation techniques such as kriging, inverse distance weighted, natural neighbor, radial basis function, and spline are used for the acquisition of noise maps (Harman et al. 2016; Gheibi et al 2022; Nasser et al. 2023; Princess Okimiji et al. 2023). Noise maps are graphical representations of the spatial distribution of sound levels in a given area and provide an effective method for evaluating urban noise. Noise maps serve as a crucial resource for planning strategies aimed at reducing noise pollution (Pandya 2003; Licitra and Ascari 2014; Oyedepo et al. 2019; Arani et al. 2022; Kumari et al. 2023).

Widespread infectious diseases and events like natural disasters or large-scale emergencies result in restrictions on people’s pursuits and recreational activities. One example includes the “Coronavirus (COVID-19)” pandemic, which is recognized as the most severe global health crisis of the current century. The disease was first observed in the city of Wuhan, China, in December 2019 (Chen et al. 2020; Jones 2020; Wang et al. 2020). Subsequently, the WHO officially declared it a pandemic (global epidemic) on March 11, 2020 (Cucinotta and Vanelli 2020; Sülkü et al. 2021). While the global spread of the coronavirus continued, the initial case in Turkey was recorded on March 10, 2020 (Cakir 2020; Çalışkan et al. 2022). In alignment with global strategies, this situation revealed the need to implement some extensive measures and impose restrictions at various scales in Turkey to prevent the spread of the pandemic. Consequently, limitations were imposed on various sectors, particularly in areas such as travel, and as a natural outcome of this process, the tourism sector was directly affected (Rivas Ortega et al. 2021; Urfa et al. 2021; Rita et al. 2023). In this context, the city of Antalya, a prominent tourism hub in Turkey with its natural and cultural endowments, has also been negatively affected by this situation. During the summer months of June, July, and August, the number of visitors to Antalya reached 6,178,419 in 2018 and 7,291,753 in 2019. However, due to the impacts and restrictions of the COVID-19 pandemic, there was a significant decrease in 2020, and the number of visitors dropped to 1,046,559 (Türob 2020). However, the lifting of COVID-19 lockdowns in Turkey has resulted in an increase in tourism activities, as of July 1, 2021. Thus, Antalya’s Konyaaltı Beach, recognized among the world’s important shores, became intensively used again. Additionally, the negative developments between Russia and Ukraine have particularly caused a significant influx of people and vehicles from the relevant regions to Antalya. Within the scope of tourism activities, the increasing number of vehicles in Antalya has also contributed to the escalation of noise pollution. In this context, stemming from events that manifest periodically, it becomes crucial to thoroughly address the potential impacts of noise pollution on the quality of urban life. Additionally, it becomes a necessity to evaluate the compliance of sound with legal regulations and the implementation of effective preventative measures to ensure that sound levels remain within the boundaries of legal parameters.

This study aims to determine the level of noise pollution experienced in recreational areas with the increasing domestic and international tourism activities after the COVID-19 pandemic, to produce spatial distribution maps of noise pollution, and to develop strategic planning suggestions for reducing noise pollution in line with the results obtained. In this context, the renowned Antalya-Konyaaltı Beach Recreation Area, one of Turkey’s prominent international tourism destinations, was determined as the study area. Noise models were produced based on national and international noise legislation, and prevention/reduction suggestions were developed for the detected noise pollution and its sources.

## Materials and methods

### Study area

Turkey is surrounded by seas on three sides, possessing coastal cities with distinctive historical and geographical features. One such city is Antalya, situated in the south of Turkey, within the boundaries of the Mediterranean Region, and stands out as an important center for both agriculture and tourism. According to the data of the Turkish Statistical Institute for the year 2022, the population of Antalya province is recorded as 2,688,004. Furthermore, owing to its roughly 657 km long coastlines, Antalya experiences a serious increase in population density as a result of increasing tourism activities, particularly in the summer months (Ozcelik and Sarp 2018; Tan et al. 2021). Nevertheless, in the summer of 2020, there was a 77.7% decrease in Antalya’s tourism density due to the restrictive measures imposed during the COVID-19 pandemic (Association of Turkish Travel Agencies 2020). Following the removal of pandemic restrictions in July 2021, the previous tourism intensity was reached again by the region. The coastal areas of Antalya have a Mediterranean climate type, characterized by hot and arid summers and warm and rainy winters. The average temperature in summer is between 30 and 34 °C, while in January, the average temperature varies between 9 and 15 °C. The average relative humidity in the province is around 64% annually. On average, 40–50 days of the year are overcast and rainy. Antalya is one of the rare regions where tourism activities can be carried out 12 months of the year with its meteorological features (Antalya Metropolitan Municipality 2021; The Turkish State Meteorological Service 2021).

**Fig. 1:**
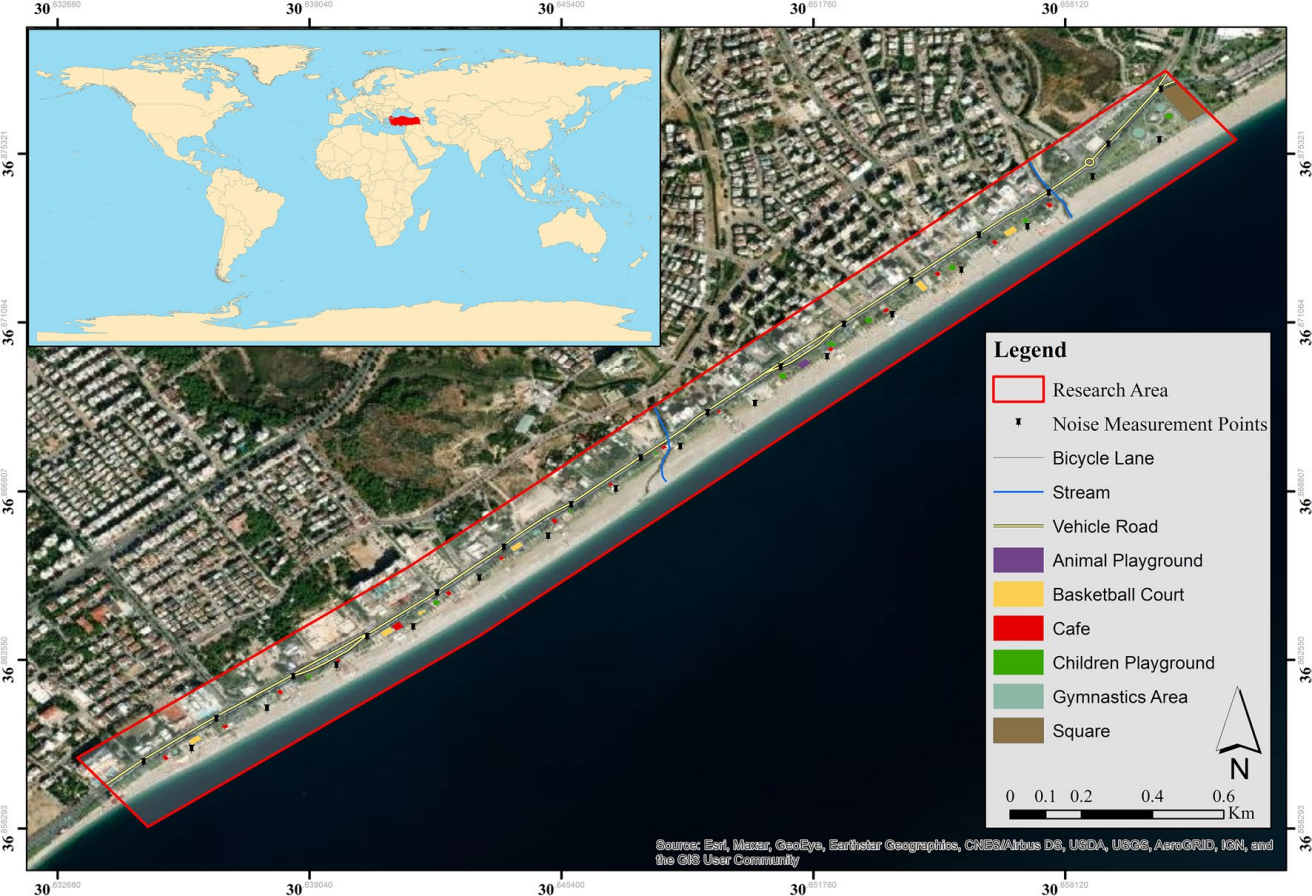
Location of study area and distribution of noise measurement points

Konyaaltı Beach Recreation Area, which was designated within the scope of the study area (Fig. 1), is located within the boundaries of Konyaaltı district in the west of Antalya. Konyaaltı’s coast is approximately 7.5 km long (Dipova 2016), and ranks among the important tourism and recreation milieu of the region and nation (Yiğit et al. 2022).

### Data sets

ArcGIS basemap image was used as the base map of the study. Then, the devices used for noise measurement were calibrated and made ready for use. Two identical PCE-322A devices were used in tandem. The devices have a measurement range of 30 dB to 130 dB and their sound measurement sensitivity is ± 1.4 dB (Table 1). The other dataset used for analysis was the coordinate values of the measurement points. A database was created by transferring the coordinate values taken from the noise measurement points with high precision to GIS software.

**Table 1:** Technical specifications of noise/sound meter

Noise levels	Low 30–80 dB
	Medium 50–100 dB
	High 80–130 dB
	Auto 30–130 dB
Dynamic range	50 dB
Display	4-digit LCD
Resolution	0.1 dB
Accuracy	± 1.4 dB
Sampling rate	2 × per second
Frequency	31.5 Hz … 8 kHz
Frequency weighting	A and C
Time weighting	Fast (125 ms) slow (1 s)
Microphone type	Electret condenser
Operating conditions	0 … 40 °C / 32 … 104 °F, < 90% RH

The number of vehicles is another variable in the data set. The number of vehicles passing by was also monitored to determine the fluctuation in sound levels in the study area that changed based on the traffic density level at different times of the day. In this context, while the sound level at each measurement point was recorded, the number of vehicles traversing the area during the same period was determined.

### Sampling point experimental procedure for noise measurement

After the evaluations made as a result of natural, cultural, and socio-economic structure features, interviews with local governments, and field observations, 31 different noise measurement points were determined at 100 m intervals. Care was taken to ensure that these points were distributed approximately equally throughout the study area. Then, the sound measuring devices were mounted on tripods 1.5 m above the ground, according to the norms determined by the “Central Pollution Control Board (CPCB)” and the “Regulation on Assessment and Management of Environmental Noise” (Koushki 1999; WHO 2000; Baaj et al. 2001; Öden and Bilgin 2019; Kalawapudi et al. 2020). These devices are positioned at points at least 3–3.5 m away from reflective and blocking surfaces, in accordance with the relevant legal legislation. Since meteorological factors have an impact on the values ​given by the sound meter (Miškinytė and Dėdelė 2014), measurements were taken on days when the wind speed was below 5 m/s and there was no rain, and the wind noise suppressing sponge that came with the device was attached to the microphone of the devices. In the measurement, “a weighted” frequency was used to evaluate the relative loudness perceived by the human ear, and the data was recorded in the system with a sample rate of 1 s. Measurements were transferred to the computer via Sound Level Meter software, where they were monitored and evaluated in real-time (Tripathy 2008; Munir et al. 2021). Measurements were carried out in the summer (01.07.2021/31.08.2021) after the lifting of COVID-19 lockdowns. Noise measurements were made during the daytime (07:00–19:00), evening (19:00–23:00), and nighttime (23:00–07:00) periods on weekdays (Monday, Wednesday, Friday) and weekends (Sunday) under the “Regulation on Evaluation and Management of Environmental Noise”. Since a set of three 15-min measurements represents daily noise levels in urban environments (Rey Gozalo et al. 2013; Romeu et al. 2011; Morillas et al. 2021), 15 min at each point was measured by researchers. Simultaneous measurements were made with 2 sound measuring devices of the same brand throughout. With the measurements performed, minimum noise levels (*L*_min_), maximum noise levels (*L*_max_), and equivalent noise levels (*L*_eq_) (Eq. 1) parameters for the *L*_day_, *L*_evening_, *L*_night_ time periods were obtained (Princess Okimiji et al. 2023).

1 $${L}_{eq}=10\;{{\text{log}}}_{10}\left[\frac{1}{N} {\sum }_{i=1}^{N}10({L}_{eq,T})i/10\right]$$

where *N* is the number of samples in the reference time interval, (*L*_*eq*,T_) is the rating level specific sound level plus any adjustment for the characteristic features of the sound.

### Noise pollution mapping using geostatistical models

To determine the best spatial distribution model that can be used in mapping noise pollution, the noise data measured during weekday and weekend *L*_day_, *L*_evening_, and *L*_night_ time periods were digitized using the Leq parameter according to their geographical coordinates in the database created in the GIS software. The number of vehicles counted was also digitized, taking into account the locations where they were counted. Then, employing the Leq parameter data, the data of the new points were subjected to kriging interpolation, a geostatistical method that allows a more impartial and minimally variant estimation compared to other methods (Isaaks and Srivastava 1989; Tercan and Saraç 1998; Tunçay et al. 2017). Kriging is a minimum-variance, spatial interpolation method that makes predictions with the weighted values of neighboring data of the point or area to be predicted, utilizing spatial dependence models obtained from covariance or semi-variogram functions (Krivoruchko 2005). In this specific context, ordinary kriging, which is a simple and widely used approach to estimate the study variable, was preferred due to its capability to provide both prediction values and associated prediction errors (Webster and Oliver 2007; Oliver and Webster 2015; Khan et al. 2023; Vedurmudi et al. 2023). Ordinary kriging is calculated with the following Eq. 3:

3 $$Z_{OK}^\ast\left(x_0\right)=\sum_{i=1}^n\lambda_i.Z\left(x_i\right)$$

where at point ${{x}_{0}, Z}^{*}\left({x}_{0}\right)$ signifies the non-sampled value, while $Z\left({x}_{i}\right)$ represents the value of the sample for the point ${x}_{i}$, ${\lambda }_{i}$ is the weight of point *i*, and *n* indicates the total number of samples (Webster and Oliver 2007; Vedurmudi et al. 2023; Nasser et al. 2023).

In this study, co-kriging interpolation was applied to analyze the noise data along with the number of vehicles. While estimating the values of unobserved points through co-kriging interpolation, semivariogram models (Stable, Circular, and Spherical) that compute the spatial variation of regional variables were utilized (Liu et al. 2024). The co-kriging method is based on the ability to use non-detailed or incomplete secondary data and to take into account spatial cross-correlation between primary and secondary variables (Goovaerts 1997). Co-kriging was preferred within the scope of this study because it is used to estimate values at points where no observations have been made in multivariate data sets. Kriging and co-kriging interpolations were applied separately for weekday and weekend *L*_day_, *L*_evening_, and *L*_night_ time periods. The mean absolute percentage error (MAPE) was used to compare the prediction accuracy of the models obtained as a result of the analyses. MAPE is expressed in statistics as a measure of the success of a prediction method in predicting (Ahlburg 1995; de Myttenaere et al. 2016; Baykal et al. 2022). MAPE is obtained by dividing the mean of absolute errors by the actual observation values (Ahlburg 1995; de Myttenaere et al. 2016; Molla et al. 2022). The equality is expressed in Eq. 4:

4 $$MAPE=100 \frac{1}{n}\sum\limits_{i=1}^{n}\left|\frac{{y}_{i}-{x}_{i}}{xi}\right|$$

where *x*_i_ and *y*_i_ are measured and correspond to the predicted values at location *i*, respectively, and *n* signifies the total number of observations.

MAPE ranges from 0 to positive infinity; MAPE = 0% indicates an excellent model, while MAPE > 100% indicates an inferior model. However, the flaw of MAPE is that even a small quantitative error makes the calculated value enormous when the observed value is small. Therefore, the closer the value for MAPE is to 0, the higher the accuracy of the interpolation model (Molla et al. 2022). Models with a MAPE value below 10% are considered “excellent,” models between 10 and 20% are considered “good,” models with a MAPE value between 20 and 50% are considered “acceptable,” and models above 50% are considered “incorrect” (Yang and Xing 2021; Baykal et al. 2022).

### Statistical analysis

IBM SPSS and JMP software were used to determine the statistical significance levels and analyze the data obtained from both noise measurements and vehicle counts. In this context, descriptive statistics values for each variable, whether the data showed a normal distribution, skewness, and kurtosis values of the variables were computed and graphs were created. In the analyses, values between − 2 and + 2 for skewness and kurtosis were considered statistically significant to prove normal univariate distribution (Gravetter and Wallnau 2014; George and Mallery 2019). Furthermore, regression analysis was performed to determine the existence of a relationship between the traffic density, and noise intensity of the study area, and in cases where a correlation was identified, it aimed to measure the magnitude and direction of this relationship (Eq. 2).

2 $${Y}_{i}={\beta }_{0}+{\beta }_{1}{X}_{i}+{\varepsilon }_{i}$$

where *Y*_i_ denotes the *i*th response, *β*_0_ is the intercept, β1 represents the regression coefficient, *X*_i_ is the *i*th predictor, and *ε*_i_ stands for the *i*th random error.

The last stage of the study involves assessing the map creation in alignment with the spatial distribution model deemed optimal based on the acquired findings. Subsequently, strategic planning recommendations are formulated, incorporating measures and planning aligned with both national and international noise pollution regulations, as well as insights gleaned from academic literature.

## Results and discussion

### Urbanization and noise levels

Legal regulations in Turkey target noise control either directly or indirectly (Official Gazette of the Republic of Turkey 2010). However, the current legal legislation concerning noise pollution in the country can be considered inadequate on its own to effectively improve the acoustic quality of cities, as the latter depends on a multitude of factors. The main reason for the poor acoustic quality of cities in developing countries, such as Turkey, is the various confusions regarding zoning and the resulting irregular and unplanned growth. This situation causes an increase in the number of sound sources due to the lack of adequate urban planning (Zannin et al. 2001; da Paz and Zannin 2010) and leads to the concentration of construction around transportation infrastructures such as highways, train stations, and airports. Consequently, a significant portion of the urban population is exposed to prolonged periods of noise (Lee 2018; Traoré 2019; Gilani and Mir 2021). In this context, studies on strategic planning are important in improving the acoustic quality of cities (Munir et al. 2021). Studies on this subject focus on different areas. While some studies concentrate on urban traffic planning (Oiamo et al. 2018; Khomenko et al. 2020), others emphasize the design of architectural structures or facades in cities (Memoli et al. 2008; Wang et al. 2015; Montes González et al. 2018). However, it is not enough to examine only these features of urban layers in improving the acoustic quality of cities. In addition, the strategic planning of urban open green areas plays a crucial role in mitigating noise pollution and enhancing the welfare and health of urban residents (Kogan et al. 2018; Rey Gozalo et al. 2013; Morillas et al. 2021). For these reasons, Konyaaltı Beach Recreation Area was chosen as the study area, not only serving as a significant open green space for recreational activities but also as the focal point of the city where the sea and green elements seamlessly integrate.

### Noise during and after the COVID-19 lockdown

Due to the restrictions imposed on indoor spaces during the COVID-19 pandemic period, the use of urban open green spaces for recreational purposes by residents has increased (Douglas et al. 2020; Samuelsson et al. 2020; Venter et al. 2020). However, this surge was not as pronounced as during periods of heightened domestic and international tourism activities. Analyzing data from The World Tourism Organization (UNWTO) on the global impact of the COVID-19 pandemic on the tourism sector reveals a 73% decrease in the number of international tourists in 2020 (January–December). This decline emerged as a significant factor in reducing noise pollution, particularly in cities characterized by intense tourism. In this context, a study conducted in various cities around the world, such as Barcelona (Bonet-Solà et al. 2021), Dublin (Basu et al. 2021), Buenos Aires (Said et al. 2020), Madrid (Asensio et al. 2020), and Milan (Pagès et al. 2020), reveals a decrease in equivalent noise levels (*L*_eq_). Throughout Turkey, there was a very high decrease in the number of tourists in April (99%), May (99%), and June (96%), when there were pandemic restrictions (UNWTO 2021). Antalya, which is described as the capital of tourism, was also affected by the decrease in the number of tourists coming to Turkey. During this period when the density was not high, residents of the region used both the sea, the beach, and the open green areas for recreational purposes without being exposed to noise pollution and in accordance with social distance rules. However, with the start of the 2021 tourism season, many local, national, and international tourists came to the region. Especially with the lifting of COVID-19 lockdowns in Turkey on July 1, 2021, tourism activities have become more intense. These intense human activities have also caused noise pollution to increase.

### The relationship between noise pollution and the number of vehicles

**Fig. 2:**
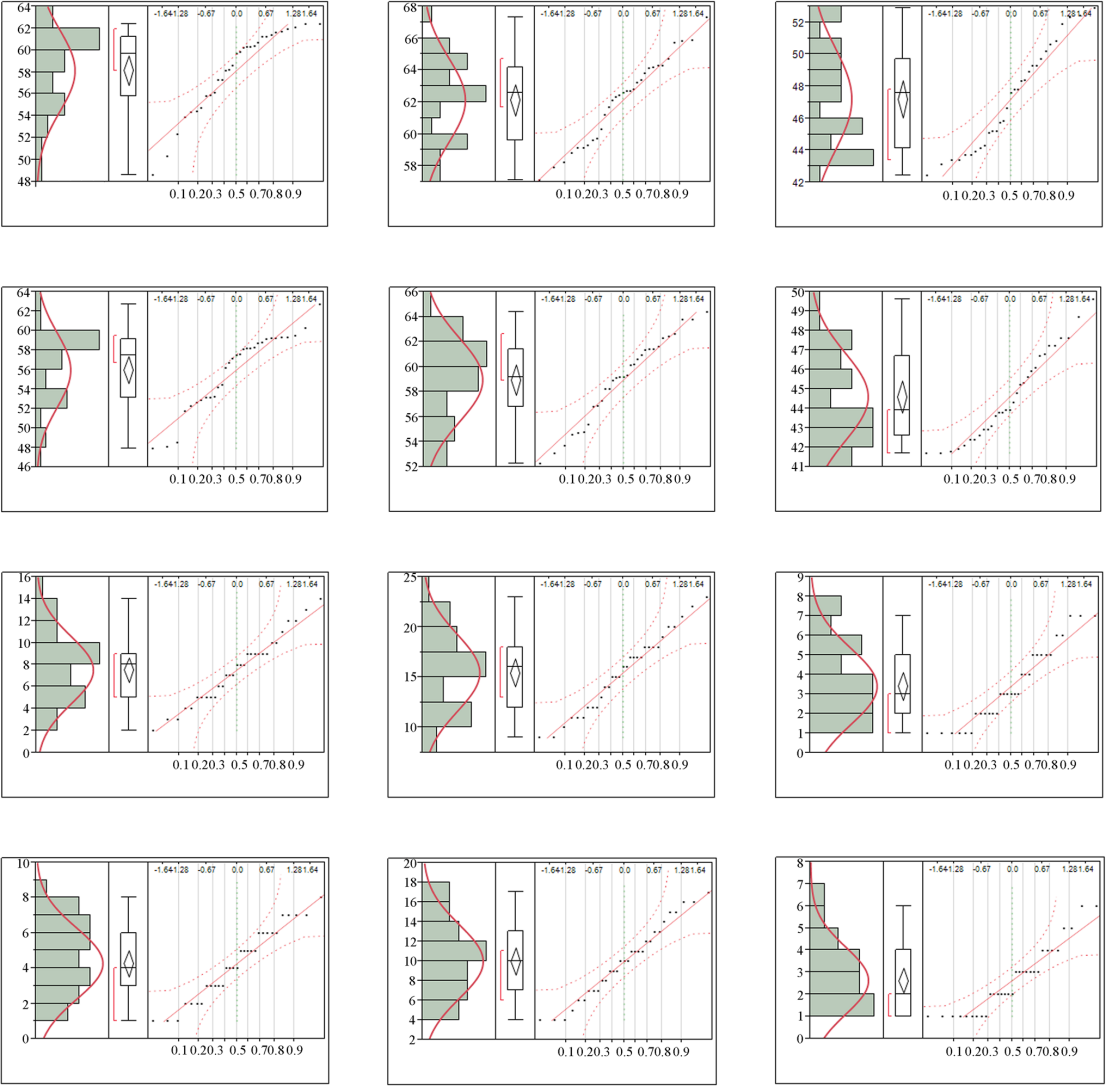
Normality test performed on the measured noise data and vehicle numbers

This study, which was conducted specifically for the city of Antalya, one of the most important tourism spots in Turkey and Europe, aimed to develop strategic planning decisions within the scope of the results obtained by focusing on the detection, modeling, and mapping of noise pollution. According to the measurement results, the normality test of the measured noise data and vehicle numbers for the weekday and weekend *L*_day_, *L*_evening_, and *L*_night_ time periods is shown in Fig. 2.

Figure 2 shows that the skewness coefficient is between − 0.928 and 0.475, and the kurtosis coefficient is between − 1.200 and 0.162. Since the skewness coefficient of the number of vehicles is between − 0.036 and 0.713 and the kurtosis coefficient is between − 1.063 and − 0.288, these data show a normal distribution. This implies that if the kurtosis coefficient has a positive value, the data shows a steeper distribution than the normal distribution, and if it has a negative value, it shows a flatter distribution (Field 2013; Tabachnick and Fidell 2013). Since the noise measurement data and vehicle number data used in the study showed normal distribution, data normalization was not performed in the analyses.

**Fig. 3:**
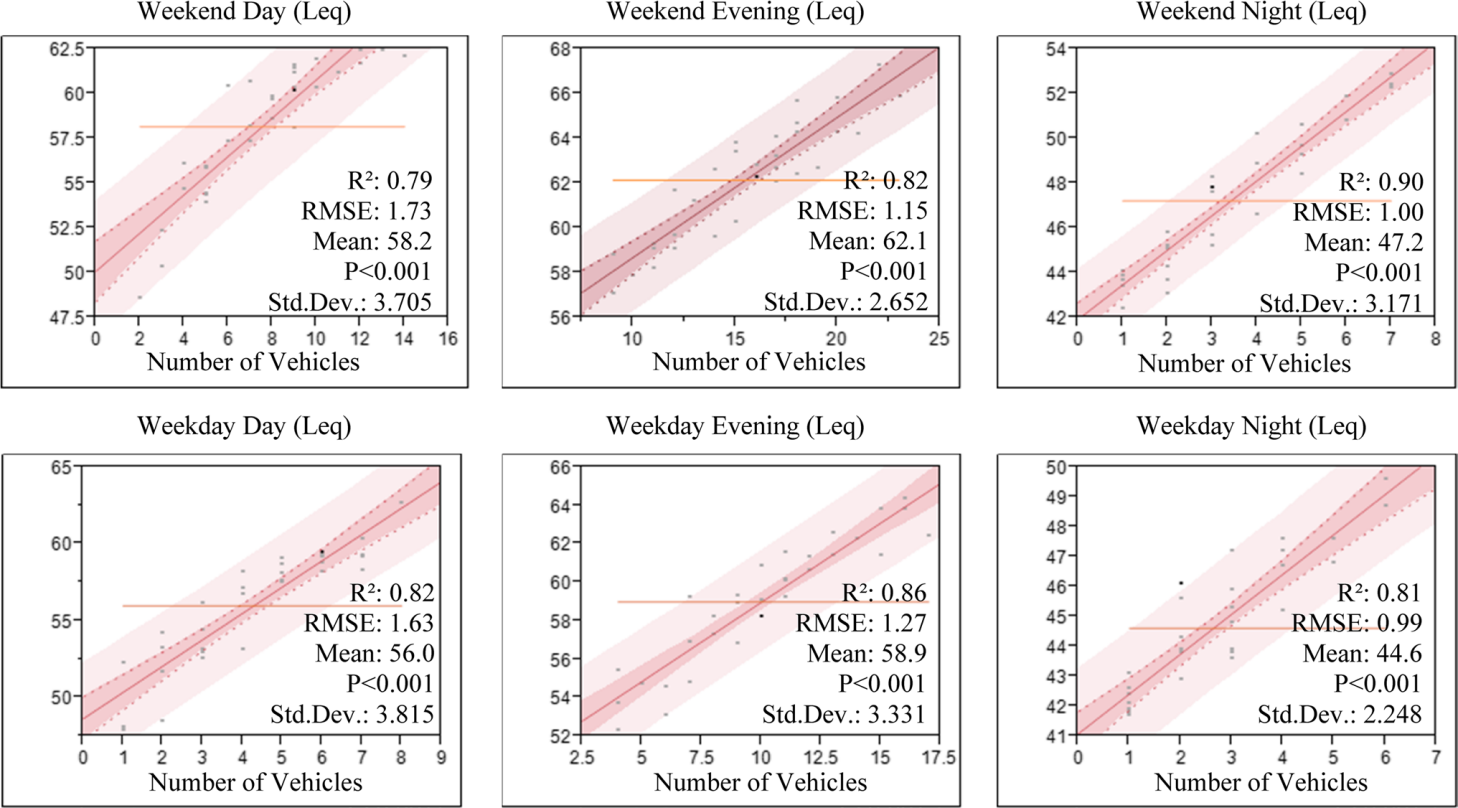
Regression analysis results of measured noise data and number of vehicles

Regression analysis was performed to determine the amount and direction of the relationship between traffic density and noise intensity of the study area, and the results are given in Fig. 3. Accordingly, it was determined that there was a high correlation between traffic density and noise intensity between 0.79 and 0.90. Although Aletta et al. (2020) and Hemker et al. (2023) emphasize that there are potentially many factors affecting noise intensity, many studies have stated that traffic density is the main factor affecting noise intensity (Čurović et al. 2021; Steele and Guastavino 2021; Asensio et al. 2020). In addition, it can be said that the correlation between weekend and night data is even higher.

### Optimal geostatistical model for noise mapping

**Fig. 4:**
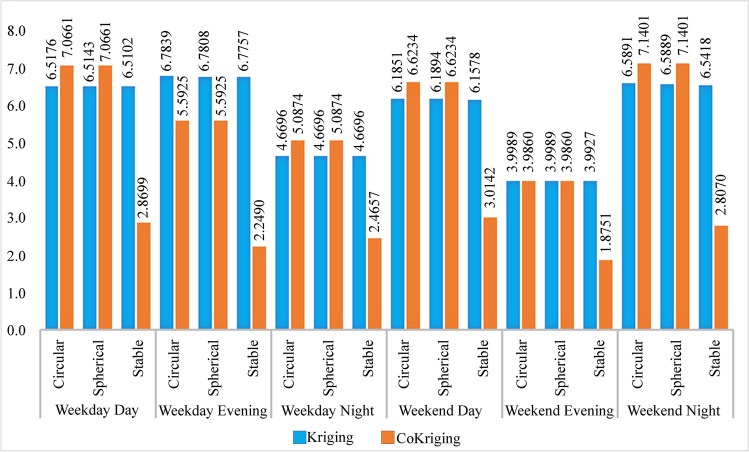
MAPE values of the models used in the analysis

In comparing predicted values with measured values (Webster and Oliver 2007), MAPE was used to evaluate all models in terms of performance prediction accuracy, and the performance of the resulting models was evaluated (Fig. 4).

**Fig. 5:**
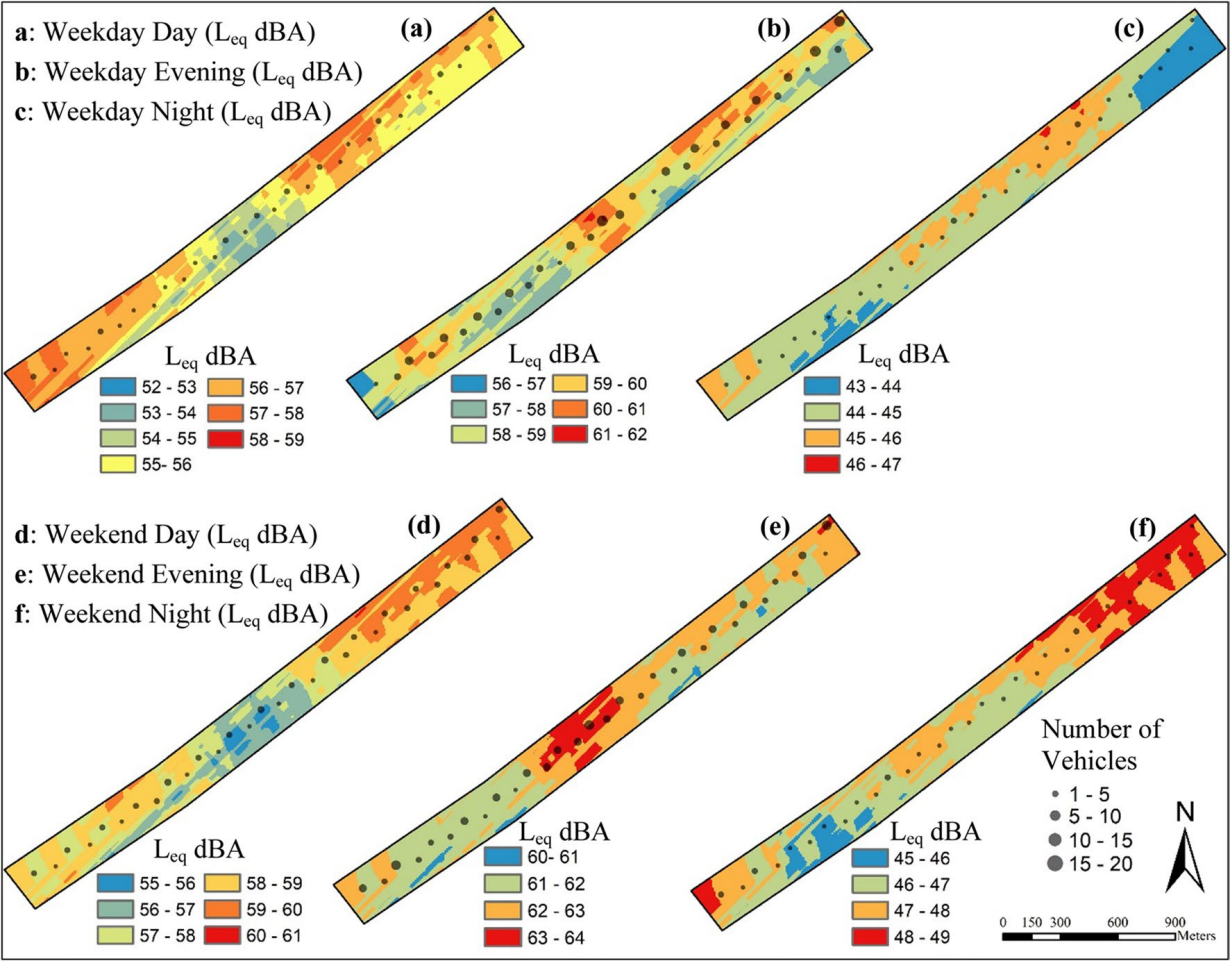
Noise map of the study area created with the co-kriging-stable model

When the data obtained as a result of MAPE analysis is examined, the performance of all models tested using both kriging and co-kriging analysis is excellent in terms of prediction accuracy (Fig. 4). However, the interpolation created with the co-kriging-stable model showed better performance than the interpolations created with other models. Because models with lower MAPE values perform better (Webster and Oliver 2007; Sajjadi et al. 2017; Yang and Xing 2021; Baykal et al. 2022; Molla et al. 2022). Therefore, the co-kriging-stable model, which shows the best performance in data estimation, was used for noise mapping. The noise map created according to this model is given in Fig. 5.

### Temporal and spatial assessment

The study area has heavy road traffic, especially during the summer season, which is the tourist season. However, today, the noise level caused by this highway traffic is not as high as in previous years. Because, with the winning project implemented as a result of the Konyaaltı Coast, Architectural and Coastal Landscaping Idea Project Competition, held in 2014 for the Konyaaltı coast, the wide double-lane vehicle road was narrowed in order to obtain more green areas and pedestrian paths. Del Pizzo et al. (2020), de León et al. (2020), and Gilani and Mir (2021) stated in their studies, in addition to reducing the flow speed of vehicles, the pavement texture and material of the road are important parameters that should be taken into consideration in reducing noise pollution. In this context, speed bumps and traffic lights were positioned along the road in the project to keep the speed of vehicles under control. This caused the vehicles to reduce their speed and thus helped reduce noise pollution caused by the road. Additionally, vehicles were tried to slow down with the help of road geometry and different road pavement materials.

Noise level measurements in the study area were made at different time periods on weekdays and weekends, as the noise level in an area varies depending on vehicle traffic density and human activities (Basu et al. 2021). The findings show that the highest equivalent noise level (*L*_eq_) in the study area is during weekdays and weekend evenings (19:00 to 23:00). Because, as stated in the study conducted by Miškinytė and Dėdelė (2014) in the city of Kaunas (Lithuania), the times of the highest traffic and pedestrian density are the times when noise pollution is the highest. Especially in this time period, the extreme temperature that is the effect of the season is less, which causes people to generally choose this region as a recreational activity and increases the rate of intensive use. Due to this intense use, the noise level is high. In addition, during this time period, the music playing in cafes and clubs along the beach, loud music coming from vehicles moving on the highway, sounds coming from children’s playgrounds and sports fields, and sounds from motorcycles used in the area increase noise pollution. The renovations carried out in the businesses along the beach, the loud communication of young people in the skateboarding and skating area, and the loud entertainments of people swimming in the sea are among the noise sources of the area.

Since weekdays (07:00–19:00) are working hours, it was observed that there were fewer users and traffic density in the study area compared to weekends (07:00–19:00). For this reason, the overall noise pollution is less during the daytime on weekdays. However, noise pollution caused by traffic can sometimes be more severe during off-peak hours than during peak hours, depending on the traffic flow speed. Because during rush hours, traffic flow speed and traffic volume decrease. Thus, engine noise and road noise caused by vehicles during rush hours are reduced and traffic noise is reduced. As seen in the data obtained from the study, the time periods with the lowest equivalent noise levels (*L*_eq_) are weekdays and weekend night hours. On the other hand, it was observed that the Lmax value was high in the measurements made during these time intervals, especially at the roadside measurement points.

According to studies on reducing noise pollution, vegetation, regardless of its type or form, is an important material in reducing the noise level and ensuring noise control (Erol 1993; Mutlu 2010; Margaritis and Kang 2017b). Studies have found that the leaves and green biomass of plants absorb acoustic energy and thus reduce noise (Samara and Tsitsoni 2011; Attenborough 2002; Van Renterghem et al. 2012; Tashakor and Chamani 2021). In reducing noise pollution, Pathak et al. (2011), it is more appropriate to use plants that do not shed their leaves and have dense green mass. In addition, this will make a significant contribution to the design in terms of aesthetics and functionality (Tashakor and Chamani 2021). When the vegetation of the study area is evaluated, it is seen that the plant population density is modest. As a result of the landscape design of the “Konyaaltı Beach, Architectural and Coastal Landscaping Idea Project Competition” implemented in the area, young and small plants were implemented in the project area. Similarly, the fact that the plants used along the driveway and walking path are young reduces the effect of the plants on reducing acoustic energy. Preferring tall and broad-leaved plant species in the revisions to be made in the work area in the future will both increase acoustic energy absorption and provide shade to the users on hot summer days. In addition, the highway and its surroundings in the study area connect many ecological focal points (city parks, urban forests, stream beds) along the east–west direction. This route should be considered the ecological corridor of the green infrastructure system on a city scale. The European Commission defines Green Infrastructure as a strategically planned network of natural and semi-natural areas with other environmental elements. This structure, designed to provide and develop a wide range of ecosystem services, is supportive of human well-being and contributes to the protection of biodiversity and climate change adaptation (Nieuwenhuijsen 2021). In addition to all these, this study area should be considered an ecological corridor within the scope of increasing human welfare, protecting biological diversity, improving mobility by providing connections between habitats, and producing ecosystem services, and should be planned as a part of the green infrastructure system (Liu and Russo 2021). This corridor, which will be defined as a result of such a planning approach, would be able to achieve many ecological benefits in addition to the essential role it plays in reducing noise pollution.

## Conclusions

After the lifting of the COVID-19 lockdowns, noise pollution has become an important problem in recreation areas due to increased user and traffic density. The findings of the study show that there is a strong positive correlation between traffic density and noise level in Konyaaltı Beach Recreation Area and that these hours constitute the noisiest time intervals since traffic and user density are highest between 19:00 and 23:00 on weekdays and weekends. In addition, it was determined that the co-kriging-stable model is the best model used in the creation of spatial distribution maps of noise and the spatial distribution maps of the noise in the study area were created with this model.

There are some limitations to this study though. One of them is that it could not be monitored the seasonal variation in noise levels since noise levels were measured during the summer period after the lifting of pandemic lockdowns in Turkey. Another limitation is that the noise measurement could not be carried out during the whole day and with more noise monitoring stations since the study was conducted with limited resources. Also, for this reason, all instantaneous variables affecting the noise level could not be included in the study. In future studies, increasing the sample size and the number of variables affecting the noise level, monitoring noise levels seasonally or periodically, and creating noise maps of certain time intervals to determine the spatial distribution of noise are recommended for a better understanding of noise dynamics in developing cities.

Overall, incentives should be provided to relevant institutions/organizations to close down motor vehicle traffic causing noise pollution at Konyaaltı Beach Recreation Area, which is an important recreational activity area of the region, and transportation systems and facilities should be built for this purpose. The recreational value of the study area will be enhanced by converting the existing vehicle road into bicycle and walking paths, increasing green areas, and planning vegetation belts between road traffic and recreation areas. In conclusion, this study is expected to contribute to researchers and decision-makers for studies to be carried out in tackling noise pollution, which varies dynamically according to urbanization and demographic changes.

## Data Availability

The datasets used and/or analyzed during the current study are available upon reasonable request.
